# The Lack of a Durable Response to Tofacitinib in Anti-PL-12 Anti-synthetase Syndrome With Progressive Interstitial Pneumonitis

**DOI:** 10.7759/cureus.49809

**Published:** 2023-12-01

**Authors:** Muaamar B Baldawi, Francis A Nardella

**Affiliations:** 1 Internal Medicine, NEA Baptist Memorial Hospital, Jonesboro, USA; 2 Rheumatology, White River Health, Batesville, USA

**Keywords:** rituximab therapy, tofacitinib, azathioprine treatment, prednisone treatment, " "arthritis, cutaneous vasculitis, interstial lung disease, s: dermatomyositis

## Abstract

We discuss a case of a 48-year-old female with anti-PL-12 anti-synthetase syndrome. She presented with dermatitis and myositis and developed rapidly progressive interstitial lung disease (ILD) while on prednisone and azathioprine, which responded dramatically to the addition of tofacitinib. However, the patient later developed arthritis, worsening skin disease, cutaneous vasculitis, and worsening ILD with corticosteroid reduction. The response to a standard dose of tofacitinib, although dramatic in the subacute phase of ILD, was not durable in terms of preventing a flare-up of skin disease, cutaneous vasculitis, arthritis, and the slow progression of pulmonary fibrosis as corticosteroids were tapered. Rituximab and nintedanib were then sequentially added to her therapy. Our experience suggests that rather than sequential addition, targeted triple therapy with tofacitinib, rituximab, and an antifibrotic should be considered early in the disease course in patients with dermatomyositis and severe ILD.

## Introduction

The anti-synthetase syndrome subtype of dermatomyositis is characterized by autoantibodies against transfer RNA (tRNA) synthetases, and it is associated with several varying clinical features such as myositis, interstitial lung disease (ILD), arthritis, Raynaud’s phenomenon, fever, and mechanic’s hands [1]. Previous reports have described the beneficial effects of the addition of tofacitinib to standard immunosuppression early in the treatment course of patients with myositis and ILD, but these findings were in the setting of limited follow-up durations of 33 days [2], 12 months [3], and six months [4]. We report a case characterized by the dramatic initial response of subacute ILD in a female patient with anti-PL-12 anti-synthetase syndrome with the addition of a standard dose of tofacitinib to prednisone and azathioprine. However, the response was not durable in the face of corticosteroid reduction, and the patient developed a flare-up of skin disease along with cutaneous vasculitis, arthritis, worsening ILD, and progression of pulmonary fibrosis requiring corticosteroid dose escalation and the addition of rituximab and nintedanib to achieve disease control. According to one report, early initiation of therapy involving the combination of tofacitinib, rituximab, and the antifibrotic pirfenidone has been employed successfully in the treatment of anti-MDA5-positive dermatomyositis with ILD observed over a period of 76 weeks [5]. Based on the aforementioned report and our own experience with the present case, we conclude that rather than sequential addition, physicians should consider the addition of targeted triple therapy with tofacitinib, rituximab, and an antifibrotic early in the disease course in patients with dermatomyositis and severe ILD.

## Case presentation

**Figure 1:**
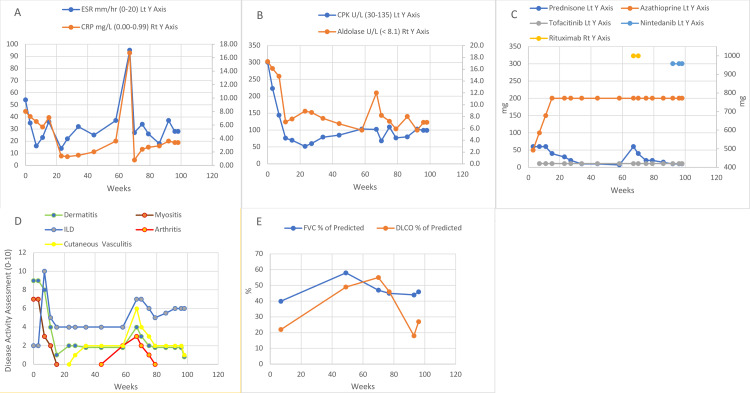
Timeline of laboratory studies, drug therapy, clinical course, and pulmonary function (A) ESR and CRP as a function of time. (B) CPK and aldolase levels over time. (C) Timeline of drug therapy and dose. (D) Course of clinical disease components and severity assessment (0-10). (E) Pulmonary function parameters of FVC and DLCO over time as a percentage of predicted CRP: C-reactive protein; DLCO: diffusion of carbon monoxide; ESR: erythrocyte sedimentation rate; FVC: forced vital capacity

The patient was a white female diagnosed with dermatomyositis at the age of 48 years (week 0) with dermatitis, Gottron’s sign, nail fold capillary dilatation, proximal upper and lower extremity muscle weakness, and crepitant fine rales at the posterior lung bases, fulfilling the European League Against Rheumatism/American College of Rheumatology (EULAR/ACR) criteria, with >95% probability, for dermatomyositis [6]. Laboratory studies revealed elevated ESR and CRP (Figure 1A), elevated CK and aldolase (Figure 1B), positive ANA with positive anti-SSA, high-level positive anti-PL-12 (anti-alanyl-tRNA synthetase), low-level positive anti-OJ (anti-isoleucy-tRNA synthetase), low-level positive rheumatoid factor, and polyclonal hypergammagobulinemia. She fulfilled the proposed criteria for the diagnosis of anti-synthetase syndrome [1].

**Figure 2:**
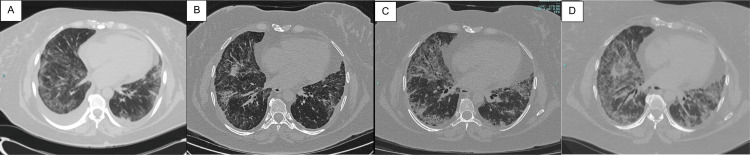
Timeline of CT chest imaging (A) CT image of the chest at week 4 revealed low-volume bilateral pleural effusion, mild pericardial effusion, and patchy areas of subpleural ground glass opacities and reticulation. (B) CT image of the chest at week 18 showed minimal pleural thickening and small pericardial effusion with mild bilateral subpleural dominant ground glass opacities, indicating improvement compared with the study at 4 weeks. (C) CT image of the chest at week 76 showed worsening patchy and confluent ground glass opacity with septal thickening. (D) CT image of the chest at week 92 showed further progression of ground glass opacities and septal thickening CT: computed tomography

The treatment began at week three with prednisone and azathioprine (see Figure 1C for a timeline of therapies). By week seven, there was a major progression of her lung disease, with shortness of breath and oxygen desaturation (89% on room air at rest; see Figure 1D for a timeline of clinical components and Figure 1E for pulmonary function parameters). She had marked lung involvement as shown on the CT of the lung (Figure 2A). Because of the rapid decline of pulmonary function on prednisone and azathioprine, tofacitinib was added to her therapy, which led to a dramatic improvement by week 11 with oxygen saturation of 97% on room air at rest and improvement in the CT lung findings (Figure 2B).

The myositis component resolved by week 15 and did not recur. Prednisone was tapered while the patient was on azathioprine 100 mg twice per day and tofacitinib extended-release 11 mg per day. At week 58, while on prednisone 7 mg per day, she began to have intermittent joint pain and swelling involving the hands and knees. By week 67, on prednisone 6 mg per day, she developed the worsening of generalized erythema, small hand joint arthritis, and worsening lung disease, and had numerous excoriated cutaneous nodules on the upper and lower extremities with a marked increase in inflammatory markers. Biopsies of the nodules at week 69 were read as consistent with neutrophilic urticarial vasculitis with subtle leucocytoclasia. Immunoglobulin and complement deposition were not found. Prednisone was increased to 60 mg per day and rituximab was initiated. The cutaneous vasculitic process nearly resolved at week 98, the inflammatory joint disease resolved by week 79, and skin erythema returned to a low level of activity. The ILD continued to be active with an increase in ground glass opacities, an increase in fibrosis, and worsening of DLCO (Figures 2C, 2D, 1E), along with the persistence of elevated inflammatory markers (Figure 1A). Nintedanib was added at week 93.

## Discussion

Several reports in the literature have described the use of tofacitinib in myositis-associated ILD [7]. Tofacitinib has been used in patients with dermatomyositis with ILD early in the disease course, but the analysis of the duration of the observed responses has been limited as none of the reports has documented a follow-up of more than 12 months [2,3,4]. The initial response with regard to the lung disease in our patient was dramatic, suggesting that the release primarily of JAK1 and JAK3-induced cytokines served as major inflammatory mediators in the lung disease at that time. However, the response to tofacitinib added to prednisone and azathioprine did not prevent a flare-up of skin disease with vasculitis, arthritis, and ILD and the slow progression of pulmonary fibrosis with corticosteroid reduction. Reinstatement of treatment efficacy with tofacitinib dose escalation to 20 mg per day has been reported in patients with anti-MDA5 antibody-positive dermatomyositis with ILD [8]. However, this was not tried in our patient. The flare-up of skin and joint disease with the continued slow progression of lung disease on prednisone, azathioprine, and tofacitinib likely indicated that higher doses of tofacitinib were needed and/or other pathologic mechanisms not adequately addressed by tofacitinib remained at play.

Rituximab has been used in the treatment of myositis-associated ILD [7]. In a retrospective study of 24 patients with anti-synthetase syndrome, rituximab was shown to improve or stabilize lung function or severity of ILD [9] but optimal benefit was not achieved until three years of therapy was completed. Our patient has had two doses of 1000 mg and further response may be expected. Early therapy with the combination of tofacitinib, rituximab, and the antifibrotic pirfenidone has been employed successfully in the treatment of anti-MDA5 positive dermatomyositis with ILD observed over a period of 76 weeks [5]. As for antifibrotics in ILD, there is high-quality evidence regarding the efficacy of nintedanib [7], and it was used in our patient.

## Conclusions

Our findings showed that while the addition of standard-dose tofacitinib to immunosuppression with prednisone and azathioprine provided a dramatic improvement in the subacute phase of ILD in our patient, it did not lead to a durable response in terms of preventing a flare-up of the underlying disease and the slow progression of pulmonary fibrosis as corticosteroids were tapered. Furthermore, our experience and the success described in the cited case report suggest that rather than sequential addition, targeted triple therapy with tofacitinib, rituximab, and an antifibrotic should be considered early in the disease course in patients with dermatomyositis and severe ILD.

## References

[1] Solomon J, Swigris JJ, Brown KK (2011). Myositis-related interstitial lung disease and antisynthetase syndrome. J Bras Pneumol.

[2] Pineton de Chambrun M, Hervier B, Chauveau S, Tandjaoui-Lambiotte Y, Combes A, Uzunhan Y (2020). Tofacitinib in antisynthetase syndrome-related rapidly progressive interstitial lung disease. Rheumatology (Oxford).

[3] Kurasawa K, Arai S, Namiki Y (2018). Tofacitinib for refractory interstitial lung diseases in anti-melanoma differentiation-associated 5 gene antibody-positive dermatomyositis. Rheumatology (Oxford).

[4] Chen Z, Wang X, Ye S (2019). Tofacitinib in amyopathic dermatomyositis-associated interstitial lung disease. N Engl J Med.

[5] Yen TH, Tseng CW, Wang KL, Fu PK (2021). Combination therapy with rituximab, tofacitinib and pirfenidone in a patient with rapid progressive interstitial lung disease (RP-ILD) due to MDA5 antibody-associated dermatomyositis: a case report. Medicina (Kaunas).

[6] Lundberg IE, Tjärnlund A, Bottai M (2017). 2017 European League Against Rheumatism/American College of Rheumatology classification criteria for adult and juvenile idiopathic inflammatory myopathies and their major subgroups. Ann Rheum Dis.

[7] Hallowell RW, Danoff SK (2023). Diagnosis and management of myositis-associated lung disease. Chest.

[8] Ida T, Furuta S, Takayama A (2023). Efficacy and safety of dose escalation of tofacitinib in refractory anti-MDA5 antibody-positive dermatomyositis. RMD Open.

[9] Doyle TJ, Dhillon N, Madan R (2018). Rituximab in the treatment of interstitial lung disease associated with antisynthetase syndrome: a multicenter retrospective case review. J Rheumatol.

